# Diagnostic Work-Up in Patients with Nonbacterial Thrombotic Endocarditis

**DOI:** 10.3390/jcm12185819

**Published:** 2023-09-07

**Authors:** Antonio Tonutti, Iside Scarfò, Giovanni La Canna, Carlo Selmi, Maria De Santis

**Affiliations:** 1 Department of Biomedical Sciences, Humanitas University, 20072 Pieve Emanuele, Italy; antonio.tonutti@humanitas.it (A.T.); carlo.selmi@hunimed.eu (C.S.); 2 Rheumatology and Clinical Immunology, IRCCS Humanitas Research Hospital, 20089 Rozzano, Italy; 3 Applied Diagnostic Echocardiography Unit, IRCCS Humanitas Research Hospital, 20089 Rozzano, Italy; iside.scarfo@humanitas.it (I.S.); giovanni.lacanna@humanitas.it (G.L.C.)

**Keywords:** non-infective endocarditis, marantic, Libman–Sacks, echocardiography, immunology, cardiac imaging, coagulopathy, antiphospholipid antibodies, anticoagulants

## Abstract

Nonbacterial thrombotic endocarditis (NBTE) is a form of endocarditis that occurs in patients with predisposing conditions, including malignancies, autoimmune diseases (particularly antiphospholipid antibody syndrome, which accounts for the majority of lupus-associated cases), and coagulation disturbances for which the correlation with classical determinants is unclear. The condition is commonly referred to as “marantic”, “verrucous”, or Libman–Sacks endocarditis, although these are not synonymous, representing clinical–pathological nuances. The clinical presentation of NBTE involves embolic events, while local valvular complications, generally regurgitation, are typically less frequent and milder compared to infective forms of endocarditis. In the past, the diagnosis of NBTE relied on post mortem examinations, while at present, the diagnosis is primarily based on echocardiography, with the priority of excluding infective endocarditis through comprehensive microbiological and serological tests. As in other forms of endocarditis, besides pathology, transesophageal echocardiography remains the diagnostic standard, while other imaging techniques hold promise as adjunctive tools for early diagnosis and differentiation from infective vegetations. These include cardiac MRI and ^18^FDG-PET/CT, which already represents a major diagnostic criterion of infective endocarditis in specific settings. We will herein provide a comprehensive review of the current knowledge on the clinics and therapeutics of NBTE, with a specific focus on the diagnostic tools.

## 1. Introduction, Methodology and Aims

### 1.1. Terminology

The term “nonbacterial thrombotic endocarditis” (NBTE) was first introduced by Gross and Friedberg in 1936, inspired by Ziegler’s initial description of sterile endocardial vegetations in 1888 [1,2]. Libman and Sacks had reported similar findings in patients with systemic lupus erythematosus (SLE) in the early 1920s [3]. From a pathogenic perspective, a key distinction is made from infective endocarditis, which occasionally leads to the use of the term “non-infective endocarditis”. However, non-infective forms encompass other conditions, such as rheumatic carditis and hyper-eosinophilic endocarditis [4]. These conditions exhibit distinct pathology, pathophysiology, and clinical manifestations, and require different treatment approaches compared to NBTE. Underlying disorders include cancer, autoimmune diseases, and thrombophilia [5], and as a consequence, various terms have been used in recent decades to describe NBTE based on these underlying features. As an example, marantic endocarditis, derived from the Greek word *marantikos* meaning “wasting away”, is used to describe NBTE specifically in patients with advanced malignancy or chronic end-stage diseases. Other variations, such as “thrombotic” and “verrucous” endocarditis, highlight specific pathological characteristics of NBTE vegetations. Additionally, “Libman-Sacks endocarditis” conventionally refers to the presence of NBTE in association with SLE and/or antiphospholipid antibody syndrome (APS) [5,6].

### 1.2. Epidemiology

Data on the epidemiology of NBTE are limited and are primarily derived from post mortem series. One of the largest autoptic studies reported an overall prevalence of 3.7%, three times higher than that of infective endocarditis [7]. The prevalence significantly increases in high-risk populations such as patients with cancer or predisposing autoimmune conditions, reaching 15% in a large echocardiographic study [8]. While the occurrence of NBTE has traditionally been considered equal between sexes [5], a slight female predominance has been observed in several reports, and is primarily attributed to the association with autoimmune diseases [9]. In a cohort of patients with antiphospholipid syndrome (APS), the prevalence of NBTE was higher in females compared to males [8], and SLE was found to be responsible for a threefold increase in thrombotic endocarditis compared to unaffected subjects [10].

The reported age at NBTE diagnosis varies, ranging from 40 to 80 years, partly reflecting differences in epidemiology, detection methods [7,9], and underlying conditions [6,7,9]. For instance, the median age at diagnosis tends to be lower in patients with NBTE associated with autoimmune disorders compared to paraneoplastic forms.

### 1.3. Methodology and Scope of the Review

The aim of this work is to provide a comprehensive narrative review of the existing data on NBTE to emphasize the significance of early suspicion, differential diagnosis, and key diagnostic and therapeutic insights. A methodical search was conducted on PubMed utilizing keywords such as “NBTE”, “nonbacterial thrombotic endocarditis”, and “non-infective endocarditis”. The selection of articles from the published literature was carried out on 20 August 2023. Given the relatively slow advancement within this field, this review transcended a mere synthesis of the current state of the art in NBTE. Instead, our objective is to underscore the multitude of unmet requirements and address the fundamental research inquiries that necessitate further exploration.

## 2. Pathogenesis and Associated Conditions

The intricate pathogenesis of NBTE involves inflammatory and thrombotic disturbances, ultimately leading to the formation of sterile thrombi composed of platelets and fibrin on the endocardial surface, without evidence of microorganisms and inflammatory cells [5]. Autoimmune diseases, malignancy, and coagulopathy represent the clinical archetypes of NBTE-associated conditions and recapitulate the underlying pathogenic mechanisms. Importantly, these three etiological categories should always be thoroughly investigated and excluded when approaching a patient with suspected NBTE [11]. Non-malignant conditions associated with systemic inflammation and wasting, such as end-stage heart failure, advanced chronic kidney disease, chronic infections (e.g., tuberculosis, HIV; particularly reflecting immunosuppression), burns, and severe sepsis, have also been observed in association with NBTE [12].

### 2.1. Neoplasm

The association between malignancy and thromboembolic manifestations is well-established, with certain histotypes carrying a higher risk, such as mucinous adenocarcinomas (e.g., lung, pancreas, breast) and lymphoproliferative syndromes [13,14,15,16]. Malignant neoplasms were the most commonly observed clinical condition in patients diagnosed with NBTE in a large observational study [9], with a notable predilection for lung, pancreatic, gynecological, and breast cancers [9,17]. Further analyses revealed that the risk is significantly higher with adenocarcinoma histology, accounting for 65% of the paraneoplastic forms of NBTE, especially in advanced stages, and metastatic cancer [17]. Despite the less clear correlation with hematologic malignancies, cases of NBTE have been described in association with acute promyelocytic leukemia [18] and T- and B-cell lymphoproliferative syndromes [19,20,21], in analogy with the other coagulation alterations commonly observed with such conditions. A thorough assessment for occult malignancy, with a specific focus on adenocarcinomas and myeloproliferative and lymphoproliferative syndromes, is thus essential in suspected or confirmed cases of NBTE.

The hyper-coagulant state associated with neoplasm may represent the *primum movens* leading to the deposition of sterile thrombotic vegetations on the endothelial surface of the cardiac valves [22], and this phenomenon was primarily observed with cardiac myxomas [23]. Malignant cancers release pro-coagulant molecules, mostly tissue factors, contributing to the hyper-coagulant state; as a paradigm, acute promyelocytic leukemia often presents with features of disseminated intravascular coagulation (DIC) [24], and the anticoagulant activity of recombinant soluble thrombomodulin has shown promising results in the treatment of such condition [25]. Additionally, the chronic subclinical inflammatory response towards the tumor, primarily driven by cancer-associated macrophages, leads to an increase in pro-inflammatory cytokines and pro-thrombotic molecules, including fibrinogen, factor VIII, and von Willebrand factor [26]. These factors drive the altered hemostasis leading to NBTE in cancer patients.

### 2.2. Autoimmune Diseases

The well-established association between NBTE and autoimmune diseases is represented by Libman–Sacks endocarditis as the cardiac involvement in patients with SLE [27]. Although valvular heart disease is not currently considered a major classification criterion for APS [28], evidence suggests a significantly higher prevalence of endocardial involvement compared to the general population [8]. From a pathogenic point of view, the deposition of antibodies in the sub-endothelial layer of the affected valves has been reported in cases of NBTE associated with APS, while the presence of immune complexes at the interface between the endocardium and vegetations has been described in Libman-Sacks endocarditis associated to SLE [29,30,31,32].

In patients with APS, there is an association between the risk of developing NBTE, the presence of lupus anticoagulant (LAC), anti-cardiolipin, and anti-beta-2-glycoprotein I antibodies of the IgG isotype, as well as the presence of two or more antiphospholipid antibodies [8]. NBTE is more frequent in patients with APS and a history of cerebral arterial events or obstetric syndrome, while an inverse association has been described in individuals with previous venous thromboembolism [8]; such correlations require further evidence but point towards the high heterogeneity of clinical APS manifestations [33]. Further research is required in order to establish whether NBTE should represent a “novel” classification criterion for APS. Moreover, the role of “non-criteria” antibodies, such as anti-phosphatidylserine/prothrombin (aPS/PT), remains to be determined, especially in the case of otherwise seronegative APS [34].

Not surprisingly, Libman–Sacks endocarditis is more frequent in patients with SLE with positive antiphospholipid antibodies, particularly triple-positive cases, whereas a negative correlation with lupus-specific autoantibodies has been demonstrated [35]. In addition, there is no established association between SLE disease activity and the risk of NBTE [35], while a correlation with neuropsychiatric lupus manifestations has been described [36,37]. These considerations highlight the need for diligent diagnostic efforts to exclude NBTE in patients with SLE and APS, especially in those with the highest serological risk profiles (two or more antiphospholipid antibodies positive) [28], as well as in the case of neurological involvement associated with SLE. It remains to be established whether new-onset NBTE may be sufficient to escalate immunosuppressive therapies in patients with SLE.

In the case of other autoimmune diseases, sterile thrombotic endocardial vegetations have been described also in patients with rheumatoid arthritis [38,39,40], inflammatory myositis [41], systemic sclerosis [42,43], Behçet’s disease [44], Crohn’s disease [45,46,47], and vasculitis, particularly with anti-neutrophil cytoplasmic antibodies (ANCA) [48,49,50,51] and giant-cell arteritis [52,53] (Table 1).

Patients diagnosed with NBTE without a definite diagnosis of an autoimmune condition are more likely to test positive for rheumatoid factors [5]. Nevertheless, the presence of serum rheumatoid factors has limited specificity, as it is frequently detected in patients with infective endocarditis as well [54]. Therefore, rheumatoid factors’ positivity cannot be relied upon as a diagnostic criterion to distinguish between infective and NBTE. Also, serum antiphospholipid antibodies have been reported in up to 14% of the patients with infective endocarditis in an observational cohort [55], and could be linked to an increased risk of embolic events in such a population [55].

### 2.3. Coagulopathy

The role of hypercoagulability in the pathogenesis of NBTE has been postulated since the first observation of DIC in up to 50% of autopsy cases [56]. Animal models have provided further support to this hypothesis, demonstrating that consumption coagulopathy is associated with NBTE in rats, likely due to abnormal production of tissue factor by sub-endocardial macrophages [57,58]. Additionally, alterations in the serum levels of coagulation factors, hypo- and hyperfibrinogenemia, and increased platelet turnover have been variably reported in patients with NBTE, especially when associated with malignancy or DIC [59]. In a large autoptic case analysis comparing infective endocarditis with NBTE, concomitant deep venous thrombosis and acute pulmonary embolism were significantly more common in the latter group, along with a higher prevalence of malignancy [60]. These findings raise the question of whether coagulopathy itself can be implicated in the formation of endocardial thrombi or whether it only represents the epiphenomenon of conditions that are likely to carry a pro-thrombotic risk (e.g., malignancy, autoimmune disorders, APS).

Currently, there are no data in the literature regarding the possible association between NBTE and classical risk factors of thrombophilia, including protein C, protein S, and anti-thrombin deficiencies; factor V Leiden G1691A and factor II G20210A mutations [61]; as well as emerging candidates like the Janus kinase 2 (JAK2) V617F mutation [62], the driver mutation of polycythemia vera-like myeloproliferative neoplasms [63]. Considering the negative correlation between NBTE and deep vein thrombosis in APS patients, it is debatable whether traditional risk factors for venous thromboembolism should be routinely screened in all patients presenting with NBTE. On the other hand, given the association between DIC and marantic endocarditis, coagulation studies should be performed in all patients with suspected or proven NBTE.

## 3. Clinical Presentation and Diagnosis

Due to the clinical heterogeneity of the condition, often presenting with established complications, the absence of systemic symptoms is directly attributable to thrombotic vegetations, as well as the usually mild valvular involvement. Post mortem examinations have historically been relied upon for the diagnosis of NBTE. When suspicion arises, the poor prognosis of NBTE and the potential fatality or disability associated with its complications make it necessary to pursue adequate tests. The diagnostic efforts should prioritize the following objectives: (i) identification and characterization of endocardial vegetations; (ii) rigorous exclusion of infective endocarditis (including cases of culture-negative infective endocarditis); (iii) screening and detection of peripheral embolic complications; and (iv) determination of the underlying condition that predisposes one to NBTE, particularly if it has not been previously identified [5,6].

### 3.1. Clinical Presentation

The diagnosis of NBTE can be challenging, as a portion of patients remains asymptomatic [64], while in over 50% of cases, clinical symptoms manifest following peripheral embolization from endocardial vegetations [65,66]. Signs and symptoms deriving from embolism represent the most common clinical presentation [10], and are significantly more frequent in NBTE compared to infective endocarditis, as non-infective vegetations are generally more friable than their infective counterparts [5]. As proof, embolic stroke manifests in approximately 20% of patients diagnosed with infective endocarditis, while it complicates a significantly higher proportion, up to 75%, of cases associated with NBTE [59]. The cerebral district is the primary site of embolization, although this observation may be influenced by the clinical significance of ischemic stroke presentation compared to other areas [67,68]. The distribution of cerebral ischemic lesions, evaluated using diffusion-weighted imaging on brain magnetic resonance, can aid in differentiating embolization from infective and non-infective sources, the last being associated with disseminated brain lesions which vary in size, whereas infective endocarditis can lead to single, cortical, territorial, or multiple strokes [69].

Additional sites of embolization include the abdominal area, i.e., the mesenteric, splenic, and renal arteries, and the peripheral limbs. In rare cases, emboli may reach the coronary arteries, leading to acute coronary syndrome [26,70]. However, despite this heterogeneity, the clinical presentation is similar irrespective of the different underlying etiologies [17,71]. Therefore, NBTE should be excluded in all patients with definite or suspected associated conditions (i.e.,: malignancy, cachexia, autoimmune disorders, or DIC), particularly when cryptogenic cerebral or splanchnic embolism is diagnosed [59]. On the other hand, whole-body assessment for peripheral embolization is mandatory in patients with non-infective endocardial vegetations.

Unlike infective endocarditis, NBTE does not cause significant damage to the valvular apparatus and is not associated with local complications such as abscesses, fistulae, or disruption of the leaflets and/or chordae [5,72]. Clinically significant valvular dysfunction is uncommon, with regurgitation being more frequent than stenosis. Left-sided heart valves are typically involved, with the mitral valve being the most common site, whereas right-sided vegetations are rare [72,73]. In general terms, the presence of a new-onset murmur or qualitative changes in pre-existing murmurs is a rare finding during physical examinations; however, when present, they should raise concerns for NBTE in the appropriate clinical context [5].

Opposite to NBTE, infective endocarditis usually manifests with fever and chills, with possible septic signs. Also, (subacute) infective endocarditis can be associated with the presence of micro-embolic phenomena such as retinal Roth spots; splinter and conjunctival hemorrhages; and immunologic manifestations presenting with vasculitic cutaneous lesions, namely, the Osler’s nodules and Janeway’s lesions [74]. Metastatic infection is also common in cases of infective endocarditis, particularly with peripheral abscesses, mycotic aneurysms, and spondylodiscitis [75,76].

Clinical features suggestive of associated underlying conditions should instead be investigated whenever a diagnosis of NBTE is suspected or confirmed. These features include general symptoms of wasting, weight loss, low-grade fever, specific organ involvement, or concomitant paraneoplastic syndromes, especially with thrombophilia or coagulopathy. Clinical hints of autoimmune diseases should also be explored in the appropriate contexts, such as the evidence of skin rashes, oral and genital aphthae, arthralgia and arthritis, Raynaud’s phenomenon, pleural or pericardial effusions, and a history of recurrent miscarriages [77,78].

### 3.2. Pathology

Post mortem histologic examination still represents the gold standard for NBTE detection. Numerous studies [12,59,60] have provided evidence that the mitral valve is particularly prone to NBTE, making it the most commonly involved site, followed by the aortic valve. Thrombotic vegetations can vary in size from less than 1 mm to 2.5 cm, can involve both the leaflets and the commissures, and are typically located on the side of low-pressure chambers, i.e.: the atrial side of the mitral valve and the ventricular side of the aortic valve [6,12,59,60,79]. Although a higher prevalence of NBTE has been reported on structurally normal valves compared to infective vegetations [60], autopsies have revealed that underlying valvular heart disease, particularly calcific or rheumatic degeneration, is also commonly present in case of thrombotic vegetations [12,59].

Sterile thrombotic vegetations represent the histological characteristics of NBTE, indicating abnormal activation of the hemostatic cascade within the affected area. Notably, such masses form in the absence of microorganisms or inflammatory cells, highlighting the noninfectious nature of the condition. Thrombotic vegetations consist of platelets enclosed and organized within a fibrin mesh, with the occasional inclusion of small amounts of red blood cells. Early signs of organization can be observed, such as the formation of granulation tissue [6,59,79]. Notably, there have been no reported histological distinctions based on the underlying comorbidity, specifically in cases of SLE/APS and cancer [79]. However, an autopsy series revealed that thrombotic vegetations associated with SLE or APS are more likely to demonstrate histological organization with granulation tissue and recruitment of fibroblastic foci, while marantic vegetations tend to undergo degenerative processes without organization [79]. Such pathological distinctions should reflect different embolic risk and require further characterization. Additionally, it remains to be determined whether signs of histological and immune-histochemical endothelitis, such as complement and immunoglobulin deposition, play a role in triggering thrombotic phenomena on the *endocardium* of patients with SLE and/or APS.

### 3.3. Laboratory and Microbiological Investigations

Laboratory investigations in patients with suspected NBTE primarily aim to exclude infective endocarditis, while in cases of autoimmune disorders, they contribute to defining the underlying condition. Additionally, coagulation studies should be performed in all patients with NBTE due to the high prevalence of coagulopathy and DIC.

To establish a diagnosis of NBTE, all infective etiologies must be ruled out, as blood culture-negative endocarditis is defined when three or more sets of blood cultures collected over 48 h remain negative despite prolonged (>1 week) incubation time [80]; collecting more than three sets does not improve the diagnostic yield [4]. Early antibiotic therapy, right-sided or device-associated endocarditis, and fastidious microorganisms are the most common causes of culture-negative infective endocarditis [4]. Blood culture-negative endocarditis may be associated with intracellular organisms (e.g., *Coxiella burnetii*, *Mycoplasma* spp., *Legionella* spp., *Tropheryma whipplei*), fastidious bacteria (e.g., *Bartonella* spp., *Brucella* spp., *Francisella* spp., *Neisseria* spp.), and HACEK group agents (*Haemophilus* spp., *Aggregatibacter actinomycetemcomitans*, *Cardiobacterium hominis*, *Eikenella* and *Kingella* spp.). Fastidious and HACEK group pathogens require specific culture media and incubation times, while cell cultures are needed for intracellular bacteria. When available, serology is a valid surrogate for diagnosis, as in the case of *Coxiella burnetii* infection [4,11]. Table 2 summarizes the most common microorganisms causing culture-negative infective endocarditis and the associated diagnostic strategies [81].

Coagulation screening, including prothrombin time (PT), activated partial thromboplastin time (aPTT), fibrinogen, and D-dimer, can reveal coagulopathy and DIC. However, it is unclear whether screening all patients with NBTE for conventional risk factors for inherited thrombophilia is an appropriate strategy [5,6]. Detection of antiphospholipid antibodies is strongly recommended in patients with NBTE, and, as per recommendations, includes LAC testing and solid-phase assays for anti-cardiolipin and anti-beta-2-glycoprotein I antibodies of both the IgG and IgM isotypes [28]. Interpretation of antiphospholipid serum assays falls outside the scope of this review and should follow the established guidelines [28].

When there is suspicion of an underlying autoimmune disease, the laboratory diagnostic workup should focus on serum autoantibodies, including antinuclear antibodies [82] at immunofluorescent assay on HEp-2 cells (ANA HEp-2 IFA), anti-double-stranded DNA antibodies [83], antibodies directed towards extractable nuclear antigens (ENA), ANCA [84], rheumatoid factors, and antibodies directed towards citrullinated peptides (ACPA). The performance quality of these diagnostic tools depends on the pre-test probability. It is important to note that patients with infective endocarditis have reported a prevalence of 24–50% RF positivity [54,85]. Antiphospholipid antibodies have also been detected in a significant proportion of patients, with the IgM isotypes possibly being associated with an increased risk of embolic events [86,87]. ANCA positivity has been reported in a small cohort of subjects with infective endocarditis, particularly directed towards proteinase 3 (PR3-ANCA) [88]. These observations reflect the prominent immunological activation that characterizes patients with infective endocarditis, as suggested by the frequent evidence of complement consumption [89]. Therefore, it is crucial to carefully exclude the presence of an infective etiology in a patient with endocardial vegetations, even in the presence of suggestive autoimmune serology. No single diagnostic test is currently available to distinguish between infective and NBTE; also, apart from antiphospholipid antibodies, no autoantibody can predict the risk of NBTE during the disease history of a patient with SLE and/or APS.

### 3.4. Echocardiography

Echocardiography remains the diagnostic standard for suspected endocarditis, with transthoracic echocardiogram (TTE) recommended as the primary imaging modality in cases of clinical concern. Transesophageal echocardiography (TEE) is recommended as a secondary investigation when TTE results are inconclusive but clinical suspicion remains elevated, and as an initial tool in the presence of prosthetic valve or intra-cardiac device infection [11]. A meta-analysis on infective endocarditis demonstrated that TEE exhibits a higher sensitivity than TTE, leading the authors to conclude that TEE should always be performed to rule out endocarditis [90]. In patients with NBTE, TEE is significantly more sensitive than TTE in detecting vegetations, particularly when small (<5 mm) [6], with a large observational study reporting a sensitivity of 97% for TEE compared to 47% for TTE [9]. As a result of these differences and despite the absence of specific guidelines, it is strongly recommended to perform TEE to exclude NBTE in high-suspicion clinical contexts, such as cases involving disseminated cerebral infarctions or peripheral arterial embolization in patients with a history of cancer or autoimmune disease.

During the echocardiographic evaluation, sterile thrombotic vegetations may manifest as either (a) small (typically < 1 cm), broad-based, hyperechoic, verrucoid nodules, usually located at the level of the valve commissure or on the low-pressure side of the leaflets [6], or (b) diffuse valve thickening. NBTE is characterized by a low-grade or lack of compromise on valve integrity [72], and patients with NBTE experience valvular dysfunction less frequently compared to those with infective endocarditis [6]. Regurgitation is more prevalent than stenosis, occurring in 54% of patients [10], and is typically of mild to moderate severity [72]. Multi-valvular involvement can occur in up to one-third of patients, commonly involving the mitral and aortic leaflets, whereas right-sided valves are generally spared [10,72,91].

### 3.5. Computed Tomography, Magnetic Resonance Imaging, and Multimodality Imaging

Data regarding non-ultrasonography imaging techniques in the diagnosis and management of NBTE are limited and are mainly represented by case reports and small case series, while most of the evidence is instead derived from infective endocarditis. These techniques include computed tomography (CT), magnetic resonance imaging (MRI), and nuclear imaging.

Cardiac CT scanning offers the highest spatial resolution compared to ultrasonography and MRI and demonstrates good sensitivity and specificity in detecting valvular lesions in patients with infective endocarditis compared to TEE [6,11], while also assessing valve thickness, mobility, and the degree of calcifications, thus overcoming one of the most important limitations of TEE, represented by calcific valve degeneration [92]. However, the temporal resolution of cardiac CT may limit the detection of small and mobile vegetations during certain phases of the cardiac cycle [92,93]. These vegetations are frequently associated with NBTE, as is the case of Libman–Sacks verrucae. Nevertheless, one of the significant advantages of a CT scan is the ability to extend the examination into a panoramic full-body view, which can simultaneously detect the presence of endocardial vegetations and peripheral, cerebral, and coronary embolization, as well as screen for malignant lesions [92].

Cardiac MRI has gained importance in recent years as a technique for evaluating cardiac morphology and function, particularly in non-ischemic and inflammatory myocardial diseases [94]. Moreover, MRI now represents the gold standard technique for the evaluation of heart valves, especially in the case of regurgitation, as it can quantify their function and degree of fibrosis [95]. Despite not being mentioned in the latest guidelines of the European Society of Cardiology for the management of infective endocarditis [11], promising data have been reported in recent years. Cardiac MRI is capable of detecting and characterizing endocardial vegetations, as well as the presence of endothelial delayed contrast enhancement, suggesting endocardial inflammation even in the absence of obvious masses [96]. Such diagnostic ability could help in differentiating “purely” thrombotic forms of NBTE, such as, for example, malignancy-associated cases, from mild-inflammatory forms, as in the case of Libman–Sacks endocarditis. Cardiac MRI can also assess the presence of local complications of infective endocarditis, such as valvular lesions and endo-myocardial abscesses [92], and has demonstrated high sensitivity in detecting NBTE [97]. However, it is questionable whether the cost-effectiveness of cardiac MRI can surpass that of TEE in cases of suspected NBTE, as this condition is typically not associated with local complications, except for the possibility of mild valvular dysfunction. On the other hand, due to its high sensitivity in detecting cerebral embolisms, MRI offers the possibility to study the endo-, myo-, and pericardium (which is often inflamed in patients with SLE), as well as the central nervous system, with a single examination [92,98].

Multimodality imaging with 18-fluorodeoxyglucose positron emission tomography/CT scan (^18^FDG-PET/CT) and ^99^Technetium-labeled leukocyte single photon emission computed tomography/CT scan (SPECT/CT) represents a major diagnostic criterion for infective endocarditis, according to the most recent European and American guidelines, particularly in cases of prosthetic or intra-cardiac device infections [11,99]. Moreover, the panoramic full-body representation of nuclear imaging techniques is useful in detecting systemic embolization and complications. ^18^FDG-PET/CT and ^99^Tc-SPECT/CT can identify areas of endothelial inflammation before morphological alterations are established, thus increasing diagnostic sensitivity [100]. Limitations include lower spatial resolution (limited to >5 mm) compared to TEE or CT scans, as well as poor specificity. Identifying areas of increased glucose metabolism (^18^FDG) or leukocyte accumulation (^99^Tc-labeled leukocytes) can indeed lead to false positive results in the case of large vessel vasculitis, atherosclerotic lesions, and recent surgical procedures [11]. Additionally, the diagnostic power of ^18^FDG-PET/CT is low in tissues with physiologically high baseline metabolisms, such as the brain, kidneys, and myocardium. SPECT/CT has been shown to be effective in differentiating infective from thrombotic endocarditis in a small cohort of patients with endocardial vegetations, as it does not detect labeled inflammatory cells in sterile vegetations [101]. However, there are limited and conflicting data regarding the role of ^18^FDG-PET/CT. A case of Libman–Sacks endocarditis with increased ^18^FDG uptake was reported in a patient with SLE [102]. On the other hand, retrospective data on cancer patients indicate that sterile thrombotic vegetations should lack metabolic activity [103], suggesting that ^18^FDG-PET/CT may assist in distinguishing between NBTE and infective endocarditis in cases of confirmed endocardial vegetations. Most importantly, ^18^FDG-PET/CT improves patient management by revealing occult cancers in over 50% of cases [103]. Efforts are required in order to elucidate whether different underlying etiologies lead to distinct findings in nuclear imaging, thus reflecting a more complex and intricate pathogenesis.

### 3.6. Proposed Diagnostic Algorithm

**Figure 1:**
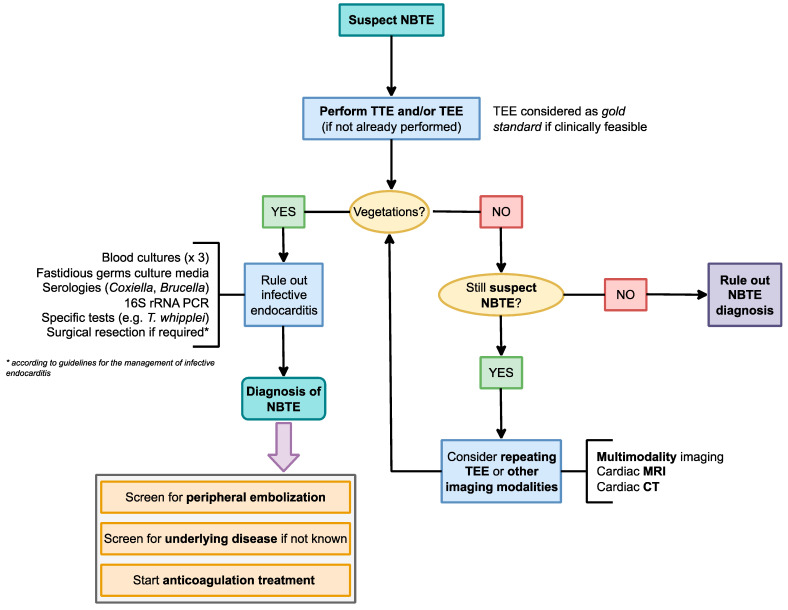
Proposed diagnostic algorithm for suspect NBTE. List of abbreviations—CT: computed tomography; MRI: magnetic resonance imaging; NBTE: nonbacterial thrombotic endocarditis; PCR: polymerase chain reaction; TTE/TEE: transthoracic/transesophageal echocardiogram.

Despite representing a neglected clinical entity, prompt suspicion and diagnosis are required when managing NBTE, particularly to rule out infective vegetations and proceed to the diagnosis of the underlying disorder, and assessment of peripheral embolization should proceed concurrently during the diagnostic work-up. A high level of suspicion remains the most important diagnostic element, and should take into account all of the aforementioned factors. A proposed algorithm outlining the key steps for suspected NBTE is presented in Figure 1. Clinical suspicion of NBTE should arise in patients with known or suspected risk factors presenting with peripheral embolization. For example, the occurrence of an acute neurological event in a patient with malignancy or SLE/APS should prompt consideration of NBTE. These patients should undergo comprehensive clinical, laboratory, and radiological evaluations to identify signs of embolization. Moreover, although infective endocarditis is more commonly linked to valve dysfunction, local complications, and hemodynamic disturbances, NBTE can be associated with valvular damage.

Endocardial vegetations may be incidentally detected during an echocardiographic evaluation performed for other clinical reasons [5]. The differential diagnosis should consider other causes of endocardial masses, such as primary valvular tumors (e.g., papillary fibroelastoma, cardiac myxoma), infective endocarditis, and Lambl’s excrescences. Clinical and echocardiographic criteria for these conditions have been proposed [104,105]. Once an endocardial mass has been identified as vegetation, the exclusion of infective endocarditis is crucial, particularly in cases of culture-negative endocarditis where ancillary laboratory and microbiological tests play a vital role [105]. No specific blood test can sufficiently and specifically detect NBTE or confirm its diagnosis without thorough exclusion of an infectious process. As previously mentioned, the results of coagulation studies, autoimmune serologies, and immune-function tests (e.g., assessment of complement fraction) are often elusive, as they rarely yield false positives in patients with infective endocarditis and are thus insufficient to identify a specific subset of patients with autoimmune-related NBTE.

When associated with cancer, NBTE can potentially offer an opportunity for earlier diagnosis [103] and better prognosis with timely treatment options. In a comprehensive meta-analysis, cancer emerged as an independent predictor of all-cause in-hospital mortality and the occurrence of embolic complications in NBTE patients, with no significant distinction between metastatic and non-metastatic neoplasms [106]. Attention should be given to the patient’s overall performance status and general conditions, which may raise suspicion of an underlying occult malignancy. These factors include weight loss, unexplained cachexia, concurrent paraneoplastic phenomena, and abnormalities in routine chemistries (such as chronic disease anemia [107], or persistent low-grade elevation of acute phase reactants not attributable to other causes [108]). The question of whether every NBTE patient should undergo an exhaustive diagnostic work-up for occult malignancy remains a topic of debate. Nonetheless, it has been suggested that patients should undergo neoplasm screening in a sex- and age-dependent manner [105]. No single diagnostic test can definitively indicate the presence of occult neoplasm in a subject with NBTE.

### 3.7. Diagnostic Unmet Needs

To identify a biomarker capable of discerning infective from NBTE, particularly among patients at high risk of thrombotic endocarditis, would be of major clinical relevance. Such biomarker could encompass tests for blood clotting times, lupus anticoagulant, solid-phase antiphospholipid antibodies, autoantibodies and assays associated with SLE or other connective tissue diseases, and, potentially, tumoral markers. However, considering the limited sensitivity and specificity of all these tests, especially when conducted individually or in the absence of high clinical suspicion, careful selection of the target population becomes of utmost importance. A composite index should incorporate echocardiographic peculiarities (e.g., dimensions and position of the vegetations) along with the pertinent clinical features. This tool could further categorize patients with (suspected) endocardial vegetations according to a high, moderate, or low risk for NBTE. The proposed questions aiming to identify potential red flags in this intricate differential diagnosis are outlined in Table 3, and should serve as straightforward reminders to guide the diagnosis even in the absence of gold standard techniques. This scenario is particularly relevant in low-income countries where NBTE is prevalent. Efforts should be addressed to define classification criteria for NBTE.

Whether the diagnosis of NBTE in a patient with an established underlying condition should prompt physicians to investigate additional predisposing factors is yet to be determined. Patients with SLE, for instance, carry a higher risk of malignancy compared to the general population [109], while a notable prevalence of antiphospholipid antibody positivity has been observed in various types of cancers and is correlated with an increased risk of malignancy-associated thrombotic events [110,111]. Further research is thus necessary to establish whether the presence of Libman–Sacks endocarditis should raise suspicion of occult malignancy in patients with SLE and/or APS, and vice versa if patients with marantic endocarditis should be tested for antiphospholipid antibodies or other coagulation defects. These observations, which extend beyond speculation, have the potential to induce significant changes in treatment strategies and prognoses.

## 4. Therapeutic Approaches

The treatment approach for NBTE is based on several aspects. First, it is important to address the underlying condition that contributes to NBTE. Second, efforts should be made to prevent the peripheral embolization of endocardial vegetations. Third and last, any associated valvular dysfunction should be corrected [11,99]. Given the lack of specific guidelines and limited evidence, which is mostly derived from case reports and series, the management of NBTE should involve a dedicated multidisciplinary “endocarditis team” consisting of imaging cardiologists, radiologists, nuclear medicine physicians, cardiac surgeons, infectious disease specialists, oncologists/hematologists, specialists in coagulation disorders, and internists/rheumatologists [6,112,113].

Unless contraindicated, anticoagulant therapy should be initiated in all patients promptly upon the diagnosis of NBTE [6,11,99], but special attention should be given to the risk of hemorrhagic infarction in patients presenting with embolic ischemic stroke [114]. Striking the right balance between thrombotic and hemorrhagic risks is crucial, particularly in patients with *stigmata* of coagulopathy [115]. In the absence of sufficient data on the use of oral anticoagulants, heparins, including both unfractionated (UFH) and low-molecular-weight heparin (LMWH), are preferred [6,113]. Vitamin K antagonists (VKAs) may be less effective than LMWH in patients with marantic endocarditis associated with malignancy; thus, heparin-based products have been the standard treatment for cancer-associated venous thromboembolism for many years [116]. However, promising evidence exists for the use of direct oral anticoagulants (DOACs) in the treatment and secondary prevention of thrombotic complications in cancer patients [117,118]. Further research is needed to evaluate the role of DOACs in paraneoplastic NBTE. Conversely, DOACs have been associated with an increased risk of thromboembolic events in patients with APS compared to VKAs (target INR 2–3), and their use is not recommended by the current guidelines [28]. Recent evidence, however, suggests that DOACs might be considered for venous thromboembolic events in APS patients without the antiphospholipid “triple positivity” profile (i.e.,: LAC, anti-cardiolipin, and anti-beta-2-glycoprotein I positivity), which is associated with the highest risk of thrombotic events [28,119]. Studies investigating different antithrombotic treatments for NBTE are warranted, and the role of DOACs should be clarified for both cancer-associated and autoimmune-related forms, particularly APS and SLE, with and without associated antiphospholipid antibodies.

Determining the optimal duration of anticoagulant therapy is unclear, and lifelong anticoagulation is recommended in cases of venous thromboembolism in patients with advanced malignancy [117], but if the cancer is cured, long-term treatment appears to be unnecessary [120]. Patients with APS-associated thromboembolic events should receive indefinite antithrombotic therapy [28]. Similar approaches could be considered to define the duration of anticoagulant therapy in NBTE patients. Furthermore, more evidence is needed to determine whether the resolution of thrombotic vegetations on TEE is associated with a significantly lower risk of embolic complications, allowing for the discontinuation of anticoagulation after an appropriate treatment period. In this regard, reduced-dose DOACs (i.e.,: rivaroxaban 10 mg *qd*, apixaban 2.5 mg *bid*) have already been approved for long-term secondary prophylaxis of venous thromboembolism [121,122]; their role could be explored in cases of resolved NBTE with persistent, longstanding risk factors for relapse (e.g.: advanced, incurable cancer).

Unlike infective endocarditis, there are no established indications for surgical intervention in NBTE patients, and decisions should be made on a case-by-case basis [11,99]. Cardiac surgery may be considered in cases of acute heart failure, severe valvular dysfunction, presence of large mobile vegetations at high risk of embolization, or recurrent embolic events despite adequate anticoagulant therapy [5,113]. Diagnosing and treating the underlying condition is crucial, although it remains unclear whether these steps contribute to preventing NBTE relapse. For example, conflicting data have been reported on the association between SLE disease activity and the risk of Libman–Sacks endocarditis [35,123]. Additionally, the role of immunosuppressive treatment in preventing the formation, progression, and complications of thrombotic endocardial vegetations in patients with autoimmune diseases requires further investigation.

## 5. Concluding Remarks

NBTE is a rare, potentially severe condition that requires prompt diagnosis and management since it is associated with underlying malignancy and autoimmune disorders. Elucidating the pathogenesis of NBTE linked to different diseases is a major area of research. Distinguishing NBTE from infective endocarditis, particularly in culture-negative forms, remains a challenge of the utmost importance, and more accurate and sensitive diagnostic strategies are warranted. While TEE currently represents the diagnostic standard, other imaging techniques hold promise as adjunctive tools for early diagnosis and differentiation from infective vegetations, including cardiac MRI and ^18^FDG-PET/CT, the latter already representing a major diagnostic criterion of endocarditis in specific situations. However, NBTE is often a neglected entity; this is reflected by the lack of specific guidelines and underscores the necessity of a holistic and multidisciplinary approach to the entire management process, from clinical suspicion to vegetation detection; differential diagnosis; comprehensive work-up, including the search of underlying conditions; and treatment. Collaborative efforts among various specialists are essential in order to optimize the patient care and outcomes involved in this complex and multi-faced condition.

## Figures and Tables

**Table 1:** Autoimmune diseases that have been associated with an increased risk of NBTE.

**Established Associations**
Systemic lupus erythematosus
Antiphospholipid antibodies syndrome
**Reported cases**
Other connective tissue diseases
Systemic sclerosis
Inflammatory myositis—antisynthetase syndrome
Rheumatoid arthritis
Systemic vasculitis
Giant-cell arteritis
ANCA-associated vasculitis (granulomatosis with polyangiitis, microscopic polyangiitis, eosinophilic granulomatosis with polyangiitis)
Behçet’s disease
Inflammatory bowel disease
Crohn’s disease

**Table 2:** Principal microorganisms causing blood-culture-negative infective endocarditis.

Microorganism	Frequency	Proposed Diagnostic Tests
*Coxiella burnetii*	37%	Serology Surgical sample: histology, immunohistochemistry, PCR
*Bartonella* spp.	12%	Blood cultures Serology Surgical sample: culture, PCR
HACEK group	2–3%	Blood cultures Surgical sample: culture, PCR
*Tropheryma whipplei*	2–3%	Surgical sample: histology, immunohistochemistry, PCR
Fungi	1–2%	Blood cultures (Serum galactomannan) Surgical sample: culture, PCR (pan-fungal and *Aspergillus* spp.)
*Legionella* spp.	<1%	Blood cultures Serology Surgical sample: immunohistochemistry, PCR
*Mycoplasma* spp.	<1%	Serology Surgical sample: immunohistochemistry, PCR
*Chlamydia* spp.	<1%	Serology Surgical sample: immunohistochemistry, PCR
*Mycobacterium* spp.	<1%	Blood cultures in mycobacterial media, PCR Surgical sample: histology, PCR
No cause diagnosed	37%	Blood cultures Serologies Broad range and specific PCR on blood and tissues Surgical sample: cultures, histology, immunohistochemistry, PCR (broad range and specific)

*List of abbreviations*—HACEK: *Haemophilus* spp., *Aggregatibacter actinomycetemcomitans*, *Cardiobacterium hominis*, *Eikenella* and *Kingella* spp.; PCR: polymerase chain reaction.

**Table 3:** Red flags in the differential diagnosis of NBTE.

1. Does the patient have a history of (a) cancer or (b) systemic autoimmune disease (especially SLE or APS)? Or does the patient’s history/physical examination suggest these conditions (e.g., unexplained weight loss, symptoms attributable to specific neoplasms, history of venous/arterial thrombosis, history of recurrent miscarriage, skin rash, etc.)?
2. Has the patient presented with an episode of arterial embolization, and in particular: (a) multiple infarcts? (b) cerebral infarcts? (c) splanchnic infarcts?
3. Are endocardial masses located in the left-sided heart valves (especially the mitral)?
4. Is the patient afebrile? Does the patient have any other sign or symptom of infection (e.g., persistent low-back pain suggesting spondylodiscitis, meningeal or neurological signs suggestive of central nervous system dissemination of microorganisms)?
5. Does the patient manifest any signs that may suggest infective endocarditis, e.g., Roth’s spots, Janeway’s lesions, Osler’s nodules, periungual microhemorrhages?
6. Has the patient ever tested positive for antiphospholipid antibodies or connective tissue disease-associated autoantibodies?

## Data Availability

Not applicable.
